# Explicitly sexing health security: analysing the downstream effects of Panama’s sex-segregated COVID-19 disease control policy

**DOI:** 10.1093/heapol/czac006

**Published:** 2022-01-27

**Authors:** Clare Wenham, Nelva Marissa Arauz-Reyes, Daniela Meneses-Sala, Corina Rueda-Borrero

**Affiliations:** Department of Health Policy, London School of Economics and Political Science (LSE), Houghton Street, London WC2A 2AE, UK; Centro Internacional de Estudios Politicos y Sociales (CIEPS), Panama City, Panama; Department of Gender Studies, London School of Economics and Political Science (LSE), Ciudad del Saber, Edificio 239, Houghton Street, London WC2A 2AE, UK; Department of Gender Studies, London School of Economics and Political Science (LSE), Ciudad del Saber, Edificio 239, Houghton Street, London WC2A 2AE, UK

**Keywords:** COVID-19, Panama, gender, health security, trans community

## Abstract

In response to COVID-19, Panama implemented a sex-segregated lockdown policy whereby women were allowed to access essential services on Monday, Wednesday and Friday and men on Tuesday, Thursday and Saturday. The logic was to reduce disease transmission by controlling population circulation at any one time. We sought to understand the impact of this policy approach on Panamanian society. To do so, we undertook key informant interviews with representatives from groups of society that have been significantly affected by this policy across Panamanian society. Framework analysis was undertaken on interview transcripts to identify key trends, which were latterly triangulated with academic, media and grey literature. Firstly, we engage with intersectional analyses to show that those most affected were marginalised groups including trans population, disabled groups, indigenous groups and migrants who faced discrimination as a consequence of this policy. Secondly, we highlight practical tensions that individuals faced relating to access to resources (financial, health-related and beyond), and third we interrogate the methods used to enforce this policy, and the role of the police and exemption passes. We conclude that this policy was regressive in that it affected those most vulnerable in Panamanian society, entrenching existing inequalities. Before implementing sex-segregated policies in future health crises, governments must seek advice of gender and equality advisors and ensure impact assessments are undertaken to understand the burden such policies may pose across society.

**Key messages**

- The costs of this sex-segregated policy were not shared equally across Panamanian society but was borne on those already most marginalised: those who do not identify in binary sex terms, those who do not have a national ID card, including indigenous groups and migrants, and created multiple barriers for disabled individuals, those in rural areas, and those with childcare responsibilities (women).
- The policy also created hurdles for accessing resources, including healthcare, finances and food for some groups. The enforcement by the police also created tensions, notably with increased sense of power resting in individual officers.
- We suggest that this policy is discriminatory, and push governments to ensure equality impact assessments and broader engagement with social consequences of policies introduced during health emergencies.

## Introduction

In March 2020, to curb the spread of COVID-19 across the population, Panama closed non-essential business to enforce social distancing and latterly implemented a state-wide lockdown, with businesses, recreation and schools closed, and the population asked to stay home, unless they were essential workers (see Table 1). This mirrors many global policy responses to eliminate, reduce or delay COVID-19’s spread (Han *et al.*, 2020). COVID-19 offers rigorous opportunities for analysis of policy variance to understand what has/has not worked, what the effects are of different policy interventions and what may be useful policy lessons for the future (Forman *et al.*, 2020).

**Table 1.** Mobility related policies in Panama during 2020 COVID-19 emergency

Policy	Institution	Objective
Executive Decree No. 490 17th March 2020	Ministry of Health	A curfew is declared across the Republic of Panama from 9 pm to 5 am, with the exception of those public and private entities providing essential services.
Executive Decree. 81 20 March 2020	Ministry of Work and Labour Development	Contracts of workers in companies whose operations have been closed will be considered suspended for all labour purposes from the date that the closure was ordered, with prior authorisation from the Ministry of Labour. This means that workers are not required to provide services and the companies pay salary. The decree states that the workers who have had their work contract suspended will be included in the list of beneficiaries of social support established by the Executive branch to mitigate the loss of regular income, whilst the suspension lasts.
Executive Decree 505 23rd March 2020	Ministry of Health	Given the increase in cases and deaths, the curfew previously imposed at the national level is extended from 5 pm to 5 am.
Executive Decree No. 507 24th March 2020	Ministry of Health	The curfew is extended throughout the national territory, 24 h a day, from 5:01 on 25th March 2020: public and private providers of essential services are exempt; only one person can leave each house, and those with chronic illness going to medical facilities can be accompanied by one other. At the same time, the decree prohibits the distribution, sale and consumption of alcoholic beverages throughout the national territory, whilst the state of emergency is maintained.
Resolution No. 1404-A 27th March 2020	Ministry of Education	Distance education, in all modalities, is authorised temporarily, targeting teachers, students and administrators in both public and private education centres across the country.
Resolution No. 360 30th March 2020^a^	Ministry of Health	New measures are adopted to restrict the mobiltiy for people, using sex and national ID card for nationals and sex and passport number for foreigners as the basis for this. Those of female sex can circulate at the indicated hours according to the last digit of their ID card or passport on Monday, Wednesday and Friday, whilst those of masculine sex can on Tuesday, Thursday and Saturday. Moreover, mobility is prohibited on Sunday, apart from health reasons. In the case of those elderly people over 60 years and those with disabilities, they are allowed to circulate, regardless of their ID card number from 11am to 1pm, under the same sex-segregated parameters of the week, and they can be accompanied by one person when necessary, regardless of this person’s sex.
Resolution No. 492 6th June 2020	Ministry of Health	As 360, but applied to the Provinces of Panama and Panama West
Executive Decree 1078 11th September 2020	Ministry of Health	The sex-segregated mobility of citizens is lifted as of Monday 14th September, leaving total quarantine in the provinces of Panama and West Panama and night curfews in Panama, West Panama, Colon Chiriqui and Bocas del Toro.
Executive Decree 1222 23rd October 2020	Ministry of Health	The total quarantine measures for weekends and the 11 pm–5 am curfew remain in place across the national territory.

^a^ The sex-segregated policy was lifted on 11th September 2020, but latterly reinstated from 28th December 2020 until 14th January 2021.

One variable for analysis is that Panama introduced sex-segregation mobility limitations as part of their lockdown (Republica de Panama, 2020a). The policy stated:

*People are allowed to circulate at the given hour according to the last digit of the ID card or passport, with those of female sex permitted on Monday, Wednesday and Friday, whilst those of male sex on Tuesday, Thursday and Saturday. On Sunday, no one can circulate in public, other than for health reasons* (Republica de Panama, 2020a).

Individuals were only allowed to seek essential services for a 2 h window, dictated by the last number of the national ID card (e.g. those who’s ID number ends in a 2 can go to a supermarket between 9 and 11 am), and on alternate days according to their sex. Such policy was thought to reduce population circulation, thus limiting disease transmission (Republica de Panama, 2020a). The male/female divide was deemed to be easier to enforce (i.e. the police could see at a distance if someone was a man/woman), which supported COVID-19 safe efforts to ensure physical distancing (Rodríguez Rondón, 2020). Importantly, this policy was linked to biological sex, expressly using ‘female sex’ (sexo feminino) and ‘male sex’ (sexo masculino), rather than gender self-identification: woman (mujer) or man (hombre).

The sex-segregated policy impacted women’s mobility more than men’s (Woskie and Wenham 2020). However, we sought to understand the broader social impact of this sex-segregated mobility policy. We know that most public policies can lead to unintended downstream effects, which often are disproportionately gendered (Sochas *et al.*, 2017; Daffe, 2019; Delamou *et al.*, 2017; Harman, 2011; Davies and Bennett 2016), and that policies which assume that all people in society will be affected in the same way will further compound exclusion and inequalities for the most vulnerable (Bowman *et al.*, 2013; Radcliffe, 2015; Saha and Shipman 2008; Mayer *et al.*, 2018). We hypothesised that such a policy would immediately create gendered effects across Panamanian society if men and women were limited in their interactions, with alternate days being permitted. This assumption was witnessed in a similar policy interventions in Peru and Bogota, which rapidly demonstrated the realities of gender inequality in the country, and the potential infringements on the rights of non-binary gender groups (Perez-Brumer and Silva-Santisteban 2020; Radi and Losada Castilla 2020; Rodríguez Rondón, 2020).

In this paper, we explore the societal costs of this policy intervention and demonstrate that it compounded existing social inequalities, challenged access to (health) resources, exposed further vulnerabilities to marginalised groups and highlight where the policy suffered from implementation challenges. We explore pertinent intersectional factors exposed by our data: drivers of vulnerability which intersect to (re-)produce patterns of inequality along gender, age, racial, disability, geographic, sexual orientation, etc., and which became apparent as predictors of how the policy would affect individuals. These effects must be considered during policy evaluations for future deployment of sex-segregated health emergency response plans.

## Methods

Firstly, we undertook a stakeholder mapping of the key groups affected by downstream effects of this policy. To do so, the team analysed media coverage, consulted with initial groups and engaged snowball sampling. Two of our research team are Panamanian lawyers with experience in human rights and feminist activism in the country. This allowed for an in depth understanding of the landscape of organisations including government departments, civil society groups representing those most at risk, trade union or sector representation groups, businesses which remained open (supermarkets and pharmacies), etc.

Once we had established the landscape of stakeholders affected or engaged in debates on this policy, we conducted semi structured interviews with representatives from each group. In total, 46 interviews were conducted between May and November 2020. These individuals were selected based on their role (or more usually leadership) within organisations supporting those affected by the policies introduced by government to limit disease transmission. The sample was purposive: individuals were asked based on their professional position, to conduct a key informant interview, in which we sought their expertise in their professional, rather than personal capacity. They were selected based on availability, subject matter knowledge and representation of a diversity of stakeholders (see Table 2).

**Table 2.** Types of organisations interviewed

Human Rights Organisations (e.g. focused on disabilty rights, youth rights, justice, etc.)	9
Feminist Organizations	12
LGBTIQ+ Organization	8
Trade Unions or other work-based organisations	9
Civil Servants and/or Government Officials	5
International Organizations	3
Total	46

We provided participants with a detailed information sheet, and if they consented, we proceeded to undertake a semi-structured interview, by WhatsApp or online call, recording these interviews for transcription where permitted. The information sheet provided to participants is included in Annex 1.

The semi-structured interviews sought to assess the (intersectional) impacts of the sex-segregated policy introduced in Panama to mitigate the spread of COVID-19. An outline of the interview guide is included in Annex 2. Yet, there was scope within the semi-structured interview for the participants to raise other issues they consider important or deviate from our central questions.

These interviews were transcribed verbatim, and then coded through framework analysis (a type of thematic analysis) as established by the research team (Ritchie *et al.*, 2013). This was chosen as a suitable method for systematically categorising, organising, synthesising and interpreting qualitative data, particularly with a team working remotely. This was an iterative, grounded method whereby all transcripts were read initially for familiarisation and preliminary content analysis, then a sample of 10 were deductively coded line by line—from this a coding framework was developed, iteratively refined throughout this analysis. This methodology also permitted the range of intersectional tensions created by the policy to be exposed deductively. All researchers reviewed the pilot framework to ensure consistency between application. This code framework was applied to the remaining data set, and additional codes were added to in the process, as new themes and content emerged. Coding was carried out by all four members of the research team, in continual communication to discuss and consider themes developing and correct application of the framework. Whilst doing so, we created an analytical log of decisions made to add to interpretative considerations of the data, and to ensure consistency between coders.

After this first coding, we conducted second-order analysis and (re)-grouping of the data to further interpret and refine themes, and key issues condensed to develop digestible findings for dissemination. Interviews, framework development and analysis were conducted in Spanish, and translations were undertaken and validated by at least two of the research team.

## Findings

Below we outline some of the key downstream societal effects of this sex-segregated policy.

### Vulnerable groups

As with several COVID response policies globally (Ryan and El Ayadi 2020; Bowleg, 2020), our data show that vulnerable groups were differentially impacted by the sex-segregated mobility policy.

#### Trans communities

One obvious group affected was the trans community (Rodríguez Rondón, 2020). In Panama, there is no gender identity law, and generally trans people do not have an ID that corresponds to their preferred gender identity. Under the sex-segregated policy, trans-men and trans-women were allowed out on days that corresponded with their sex at birth on their ID card, meaning they could not go out on the day determined by their gender identity. We learned many sought to undermine this and went out on the days with which they identified. As we heard:

*You can come out as a woman, and can say “I’ve lived my whole life, more than 10 years as me, and I’m a woman, but a policeman can say “ID document says you are a man”, and that’s it.* (Healthcare worker)

Whichever day they went out, this led to increased discrimination by the police, who stopped individuals to enforce the policy based solely on appearance, asking them to return home or to go out on a different day (Summers, 2020; Human Rights Watch, 2020). One trans woman was fined $50 for breaching the policy, a similar experience repeated elsewhere (Ott, 2020), having been told:

*This is the day for real women, and you are not a real woman* (Trans Group Representative B).

For some, this inability to go out in public on the day corresponding to their gender identity meant they had not left the house for many months for fear of discrimination, and others were left questioning the way they presented themselves publicly. One trans person had confided to their rights group:

*I feel unwell; I think that this doesn’t make any sense. Maybe I should leave the transition aside, and dress as a woman.* (Trans Group Representative B)

We also heard of the significant consequences of trans individuals feeling they were unable to go out on either day of the week:

*We had to take food urgently to one trans women who has a son, and they hadn’t eaten for two days.* (LGBTIQ Group Representative)

#### Indigenous groups

Indigenous groups were also disproportionately impacted by the policy (Power *et al.*, 2020). This appeared in two discrete pathways. Firstly, we heard that indigenous groups may not have been aware of the policy initially, if they did not speak Spanish well, and on top of that may not have had ID cards to be able to show police that they were out on their assigned hour:

*Men were out going to work, and they didn’t have an exemption pass – they were working, they didn’t speak the language well… and they were being detained.* (Indigenous Rights Group Representative)

This also reflects that indigenous groups are more likely to work in the informal sector (Madrid Martínez, 0000) and therefore many continued to work despite the quarantine, and were further burdened by the sex-segregated restrictions. The impact of not being able to work, or being detained, resulted in reduced income and indeed, food insecurity:

*There was no money for many [indigenous] women to buy food.* (Indigenous Rights Group Representative)

A second interconnected impact was that many indigenous people were then not able to access government food packages and social assistance:

*To be eligible for the $80, you have to be registered, and we know that the majority of indigenous groups in this country are not registered.* (Feminist Organization Representative)

A similar concern was noted about migrant populations.

#### Groups with disabilities

Disabled groups noted considerable concerns about the 2 h window for accessing essential services since this disadvantages those for whom mobilising would take longer. As one summarised:

*if your hour is at 5 pm… but for you it takes three, four or six hours to move to do a simple task because of your disability, coupled with the lack of accessibility in public spaces; the city in itself is violent for those with a disability.* (Feminist Youth Group Representative)

Thus, we heard that many disabled individuals at the start of the policy were either unable to go out or breached policy in doing so. There were also concerns as to whether carers and the disabled could go out at the same time if of opposite sex. This was notable given most carers are women and some individuals are not able to be left unattended whilst a carer were to go out to secure essential goods (Human Rights Organisation A). It was soon clarified by the government that disabled people were able to go out between 11 am and 1 pm with a carer of the opposite sex.

#### Children

Children and the care responsibilities of parents were seemingly missing from consideration of those crafting the policy, as parents were not supposed to be out of the house with those who were of the opposite sex. As we heard:

*A mother went out with her teenage son and the police stopped them because it wasn’t a man’s day.* (Representative Department of Childhood, Adolescence and Family)

The government assumption was that children:

*were supposed to stay in the home the whole time* (Representative Department of Childhood, Adolescence and Family).

There was also concern that such regimen could affect children moving between two parent households. Yet, such a mandate was a particular issue for single parents who were then unable to leave their house whilst seeking essential services. We heard that many parents sought informal childcare arrangements to overcome this, and indeed defying requirements for social distancing, or had to take their children to the supermarket, against regulation.

Moreover, those who did take their children out were further burdened by the 2 h time limit, noting that it would take longer to go to the supermarket with children in tow, than without. One interview further highlighted that this disproportionately penalised women compared to men: *‘[women] do all the additional things with children, taking them to get vaccinated, to get medical support… so this means they have much to do in the two-hour window*.’(Public Ombudsman Representative).

### Access to resources

Whilst quarantine orders globally have impacted access to resources (Ahmed *et al.*, 2020; Tang *et al.*, 2020; Carroll *et al.*, 2020), the sex-disaggregated policy in Panama amplified barriers into accessing a range of tangible and intangible resources. These questions of access affected the spectrum of vulnerable groups discussed above. We have tried to use a range of data points from different social groups to demonstrate the wide-reaching nature of these issues.

#### Access to healthcare services

Demand side access to healthcare services has been limited globally in the wake of the pandemic. However, there were particular challenges in Panama under the sex-segregated mobility policy. For those living rurally and indigenous groups, who might live 3 or 4 h from the specialist care needed, it was not possible to get to the appointment and back within the 2 h window designated to each person or challenges remained for those who were reliant on transportation from someone of the opposite sex, with many choosing not to seek care as a consequence.

Beyond this temporal limitation, the sex-segregated mobility meant obtaining appointments on the day which individuals could go out, which may have affected their care pathway. As one individual reflected:

*I have stitches in my mouth which need removing. These were supposed to be cut out on a Saturday, but I had to wait until the appropriate [female] day to get them out, which is the following week.* (Afro-Youth Organization Representative)

In theory, a healthcare appointment was an exception permitted under the sex-segregated policy, by producing a letter or email, yet this was often a barrier for those with limited resources:

*if you are someone living in a poor area, with little money, how are you going to print something that they’ve sent you online? You might have a cellphone, but no data – how can you show [the police] that they’ve sent you an email?* (Gender Expert, International Organization)

Moreover, the police checks to see if individuals were circulating per their allotted 2 h window and/or on the appropriate day and/or with a medical exemption had significant impacts on access to services for vulnerable groups:

*yesterday I heard about an event where the police took almost 40 minutes, looking at documentation, asking questions, and she [a trans woman] almost missed her appointment.* (Gender Expert, International Organization)

#### Access to Sexual and Reproductive Health (SRH) services

Access to SRH services was noted as a particular concern by many respondents on both the supply and demand sides (Tang *et al.*, 2020).

On the demand side, providers stated that they were overwhelmed on female days, with women waiting in queues outside the door, and women becoming concerned that they would not be able to access services within their 2 h window. As one stated, this impacted the service provided, trying to ensure equity in provision: ‘*we had to reduce consultation time to 5-10 minutes to allow us to get to all those waiting to be seen*’ (Healthcare provider).

And yet overall, demand for SRH services reduced (as fewer people attended on men’s days). However, there was an imbalance between how many people attended on women’s days and men’s days, and so as to fit all the women in who were waiting, they had to reduce the appointment time.

Providers also noted that the 2 h window and reduced demand for services had an impact on their turnover and in turn their continued supply provision. As fewer people were attending health clinics, this meant that some private/not for profit services found it harder to continue to offer appointments, reliant on service user payments for service delivery:

*The income reduction has been radical, barbaric, at another level, …* (Sexual and Reproductive Health Provider)

The combination of reduced access, coupled with the longer term impact on service provision is of significant concern more holistically: *‘We’re sure that we’re going to see a significant increase in pregnancies and that many of these will be completely unplanned, unwanted and will continue because there’s nothing else to do’* (Sexual and Reproductive Health Provider).

#### Access to medicines

The policy implemented by the Panamanian government created further barriers for those accessing medicine. Most notably, the limitation of the 2 h window meant return visits to providers. As one noted ‘*my roommate had to find medicine for her mother, and it took her 5 hours over three days [almost the entirety of her permitted 6 hours in 3 days] because each day she went there was a problem’* (Feminist Youth Group Representative).

This was also flagged as an issue when medication was prescribed to take the next day. In all likelihood, attending the appointment for diagnosis would have taken the 2 h on the first day, and the patient might then have to wait for two further days to access the pharmacy for medication. Notably, accessing a pharmacy did not count as an exemption to the policy in the same way that medical appointments did. Such logistics were repeated:

*Every time I went, it would have to go on two separate dates. One day to go and request the medication, and a second day to pick it up.* (Feminist Organization Representative B)

Whilst the government subsequently established a pharmacy delivery service to those with chronic conditions to try and overcome such challenges, such mechanism posed further concern for patient confidentiality. As one respondent demonstrated in the case of HIV/AIDS patients:

*It’s not as simple as here are your pills, bye… we try to camouflage the content by saying at the door “here are your diabetes pills, or here is your paracetamol” because there remains such a taboo about HIV infection, and many people are living with family members who are unaware [of their status].* (LGBTIQ Organization Representative B)

#### Access to food

Access to food was also impacted by the sex-segregated policy. Many talked of informal arrangements springing up between neighbours, particularly amongst individuals living alone, where women asked men to pick them things up from the shops on their days and vice versa.

Yet, we identified further concerns relating to food security. Social distancing requirements meant that there were long queues to supermarkets, with the result that often people were unable to do a shop within the 2 h window allocated to them.

*The idea of time is a bit absurd. You have two hours only; one hour to do what you have to do, go to the supermarket. Then you have one hour for getting there and back…. So that only really leave you one hour for going to the supermarket, and the queues*. (LGBTIQ Group Representative C)

Sex-segregated access to supermarkets created additional hurdles to lower socio-economic groups: many working in the informal sector rely on a daily income and thus daily visits to shops to purchase food. To mitigate this, the government provided food parcels to citizens, and a grant of $80 per family (which was later increased to $100), yet several respondents highlighted that these food packages and financial assistance were not enough, if they arrived at all (Republica de Panama, 2020b).

As one respondent stated *It was fortunate that behind (this one migrant’s) house there was a little mango grove, and he was able to survive eating mango in the morning, lunch and at night, because he had nothing to eat* (Trans Group Representative A).

Interestingly, farmers and farm workers were permitted to continue working as an exemption to lockdown and to the sex-segregated mobility policy to ensure food security for the nation. Yet, given the masculinised sector of work, for some respondents such exemption meant a return to traditional male ‘provider’ roles:

*Men continued to fish, hunt and farm, whilst women stayed at home with the children* (Feminist Youth Movement Representative).

More light-heartedly, the impact of the sex-segregated policy was felt amid purchasing behaviours within supermarkets, with men buying more expensive items and items on offer compared to women. Whilst this does not suggest food insecurity, it demonstrates an additional gender effect, which would have knock on effects on supply chains. A supermarket executive noted:

*on men’s days we sold more, because of the greater quantity of men who went shopping, but the average trolley costs was less for men than it was for women…. a further curious fact was that we sold more offers on men’s days than women’s’.* (Superstore chain Representative)

### Enforcement and security

Importantly, this policy was not based on individual’s self-adherence to the days of the week, but police actively enforced this stark regulation. Several stakeholders raised concerns about the role of the police in this way (Faull and Kelly 2020; Bradford *et al.*, 2020).

Police were central to enforcing the sex-segregation, stopping people out on the wrong day and requesting exemption passes. Notably, we heard that they worked mostly in pairs in the street, bringing greater perceived power to their efforts to mitigate population movement. Indeed, they were described by our participants as: *‘the public force for necessity’* (Feminist Youth Movement Representative) and a *‘population control’* weapon (Workers Union Representative). The police’s power, and indeed sanctioning efforts, were of concern to several groups of key informants:

*The police says who can go out and who cannot go out …. The police say who is detained, who can go out in the street and if you have to stay in.* (Workers Union Representative)

However, several participants voiced concerns as to the variability of how strictly the policy was enforced by the police, and when they turned a blind eye, particularly when it came to trans people and which day they were out in public. As became apparent, some police were more understanding than others, yet as a conservative force within Panama most spoke of a lack of flexibility by the police:

*[a policeman’s] religion, resolve and power decide on the application of the law.* (Feminist Organization Representative)

The policy could also be waivered with an exemption pass (permitting being out at a non-allocated time/day). Initially, these were available only to those working in essential services, yet many interviewees told that these passes soon became commodified, as one respondent discussed:

*if you had money or the right connections you can get your pass and if you are man or woman, you can go anywhere you want at anytime.* (Feminist Youth Movement Representative)

Beyond these exemption passes, several participants noted that they perceived men to be contravening the regulations more than women and more likely to go out on women’s days or not at their assigned hours, regardless of whether they held an exemption pass. As one said:

*There have been more police interventions for disobeying quarantine restrictions on men’s days than on women’s days. 80% of those breaking restrictions are men.* (Feminist Organisation C)

Here, we see that not only the police were continuing to enforce the policy, regardless of the individual’s gender, but that men appeared to be less likely to adhere to the public health guidance than women, a trend replicated globally.

## Discussion

The sex-segregated lockdown policy implemented in Panama can be seen as a regressive public policy that compounded inequalities and produced new barriers for access to essential services, disproportionately affecting already marginalised communities. Trans communities, disabled individuals, rural dwellers, indigenous groups, single parents, the poorest and women, people who in other societies are considered to have protected characteristics (Kumra *et al.*, 2012), all bore the brunt of the downstream effects of the policy, and such (predictable) impacts appear to not have been fully considered in the development of the policy. Indeed, in May 2021, the policy was challenged in the Supreme Court of Panama and was deemed unconstitutional in this forum, as it failed to guarantee the constitutional right that Panamanians have of freedom of movement and freedom of peaceful assembly (Republica de Panama, 2021).

In particular, this policy automatically continued the Panamanian lacuna in recognition of gender identity (Ryan, 2018). In doing so, it has also raised trans activism to a position of visibility previously not witnessed, as the trans community sought to challenge the policy which directly placed them unable to be in public at any time (Rodríguez Rondón, 2020; Summers, 2020; Ott, 2020; Arroyo, 2020) (women, workers and other social groups have also been involved in considerable grassroots activism). Instead, the government issued a series of advisories to the security sector to prohibit discrimination to LGBTI groups and thus placed the burden of responsibility on to these traditionally conservative street level bureaucrats, with variable implementation, and often resulting in discrimination and humiliation for those stopped and/or charged with breach of policy. As mobility was governed by sex at birth and given there is no gender recognition legislation (Ryan, 2018), it is virtually impossible for those who identify as a different gender to indicate this to authorities.

Moreover, similar policies were introduced in Peru and in Bogota (Gobierno de Peru, 2020; de Bogota, 2020). In Peru, the government retracted the policy a week after it was first implemented in April, as policymakers recognised that it was further entrenching inequality within society (Semana, 2020). In particular, the discrimination of the trans community was important enough in Peru for the President to offer clarification as to which days trans men and women could go out (EFE A, 2020) and indeed was the first time that a Peruvian president had ever expressly addressed the LGBTI community and ultimately helped contribute to the end of the sex-segregated policy in Peru. In Bogota, the mayor had sought to ensure that gender identity was recognised and that people were able to decide which day to go out on (Moreno Hernandez, 2020). Whilst open acknowledgement of the impacts on trans communities did not mitigate against some issues of continued access to resources, Peru and Bogota’s considerations of this did seek to reduce the intersectional impacts evident in Panama. Trans activism, whilst evident during 2020, had less of an effect on shifting government policy in Panama. Notably we detected in our data a lack of consensus amongst trans groups, with some seeing this policy as an opportunity for visibility and to push for greater policy space highlighting the disproportionate effect it was having on them. Yet, others feared that pushing back publicly against a public health measure, such as by going out on their non-assigned day, would paint them as ‘trouble-makers’ amongst policymakers and the public and would be detrimental to trans rights.

There were further intersectional impacts of the sex-segregated policy: For the disabled community, the lack of clarity about being out with a carer of the opposite sex reflected a failure of government to recognise their needs or to clearly communicate to those affected. Given the lack of clarity, some disabled people preferred to simply stay at home, but in doing so this reproduced memory of losing independence that they had acquired as disability rights and access have increased. Yet, this raises broader concerns in recognising the gendered nature of care, and the disruption to informal and formal support networks.

However, the problem with this policy was not just the sex-segregation, but the 2 h window to be able to access essential services was just as, if not more, restrictive than sex-segregation for those interviewed. The combination of the two was particularly challenging as it further restricted access to resources, healthcare and food, and this barrier to access was more acute for marginalised groups. We were surprised by this, as we set out to analyse the impact of the sex-segregation and found that this cannot be studied without consideration of limiting the time of people’s public participation. We are also not aware of other locations enforcing a time limit on individual’s public activity during COVID-19, other than curfews at night.

Moreover, the ability for police to undertake stop and search activities on those most vulnerable in society, such as trans individuals or migrant communities, more easily, raises further concerns of the increased securitisation of infectious disease control policy (Wenham, 2019). There are very real concerns that the increased involvement of the military and the police in the enforcement of lockdown and other disease control measures is increasing the power and reach of the security sector globally and leading to increased insecurity and violence in communities (Stott *et al.*, 2020; Pleyers, 2020). This has to be considered alongside the individual decision making of the security sector, a conservative force that may seek to implement policy in a way that restores more traditional values or those driven by religion, as seen in their interaction with trans communities in Panama. More consideration is needed to understand how such increased securitisation because of health emergencies can disproportionately target those most vulnerable within communities.

## Limitations

As with all qualitative research, this study is limited by the participation of key informants, and on the selection of those who we invited to interview. We do not claim to make generalisations and recognise individual perceptions, but hope that interviewing individuals representing organisations working with discriminated and marginalised groups (rather than as individuals) has meant we have captured a larger understanding of the effects of this policy. This study was undertaken in depths of the pandemic in 2020. Thus, participations were restricted by individual key informant’s availability, noting the increased informal labour which had befallen many across society.

Moreover, we also actively sought to interview those groups which we perceived to be affected by gendered impacts of the sex-segregated policy from our initial mapping, and thus the absence of data from those who did not consider to be at significant risk of the downstream effects of such policy may produce confirmatory bias in our findings. However, given the lack of consideration of such groups in routine research, we felt it important to detail and understand their experience of the pandemic. We also conducted the research remotely, via phone and WhatsApp. This may have limited the interpersonal relationship and freedom of discussion between interviewer and interviewee. Finally, we recognise our own positionality as female researchers/activists, two of whom were Panamanian and directly affected by the restrictions, and with our own opinions on the policy. Any unconscious bias as a consequence is unavoidable.

## Conclusion

We sought to understand the socio-economic effects of the sex-segregated policy implemented in Panama. As we have demonstrated, these were numerous, considering limitations on access to health and food; intersectional effects; and the empowerment of the police. Given the unequal effects that were felt across Panama because of this sex-segregated policy, we suggest that Panama should seek to ensure equality impact assessments of policy developments, to try and pre-empt the particular impacts that were experienced across the country and to reduce the creation of further inequalities within society. Whilst this norm of assessing potential inequalities introduced through new policies has emerged within the National Committee of Violence against Women (CONVIMU), such policy assessment has not been systematically applied across policy spaces in the country. This should not be limited only to Panama, but all governments who seek to introduce emergency measures to curb the spread of infectious disease must do so in a way that minimises the unequal effects. To do so, gender and equality advisors should be consulted, alongside other social experts to ensure that epidemiological approaches to disease control do not provoke further societal inequality and unrest. Once such analysis is conducted, policies can be further adapted to facilitate additional support for those groups most disproportionately impacted, such as the marginalised groups highlighted in this research; LGBTIQ communities, indigenous groups, the disabled for example, to ensure that additional support is provided to these groups. This could include more circulation time for those living in rural areas or with accessibility needs, or for allowing the trans community to be able to circulate by day which matches their gender identity, rather than sex on national ID card (yet in the absence of gender identity legislation in Panama, such recommendation may be unmanageable). Nevertheless, marginalised groups much be given due considerations in future pandemic response policy, which must be a lesson for all policymakers in Panama and indeed at the global level.

## Supplementary Material

czac006_Supp

## Data Availability

The data underlying this article cannot be shared publicly due to the privacy of individuals that participated in the study, in line with the informed consent form they agreed to.
