# Supplementary material for: Explicitly sexing health security: analysing the downstream effects of Panama’s sex-segregated COVID-19 disease control policy
Source: Health Policy Plan. 2022 Jan 27;37(6):728–36. doi: 10.1093/heapol/czac006 (PMC8807319; doi:10.1093/heapol/czac006)
Supplement: czac006_Supp [file czac006_supp.zip › Annex 1 blind.docx]

**Las Politicas de COVID-19 y G**é**nero**

**DECLARACIÓN DE INFORMACIÓN DEL PARTICIPANTE**

1. **De qué se trata en la investigación?**

Se le invita a participar en un estudio que examina las políticas de distanciamiento social basadas en el género implementadas por Panamá, Perú y, más tarde, Bogotá. Los lunes, miércoles y viernes, las mujeres pueden salir de sus hogares, mientras que los martes, jueves y sábados solo los hombres pueden hacerlo. Si bien la mortalidad de COVID-19 está demostrando que mueren más hombres, sabemos que las mujeres experimentan desproporcionadamente los efectos secundarios del brote; con un mayor trabajo doméstico durante el autoaislamiento, mayor cuidado de los niños si las escuelas cierran y corren el mayor riesgo de inseguridad económica debido a los contratos de trabajo precarios. Las políticas de Panamá, Perú y Bogota brindan una oportunidad única para examinar otro impulsor potencial: un mayor riesgo de transmisión de persona a persona debido a las diferencias en las prácticas de distanciamiento social y los efectos secundarios de esta política de género.

1. **Quién está realizando la investigación?**

La investigación está siendo dirigida por investigadores de XXX). El proyecto de investigación se ha sometido a una revisión ética de acuerdo con la política y los procedimientos de ética de la investigación de XXX

1. **En qué consiste la investigación?**

Para nuestra investigación cualitativa, estamos realizando una revisión de literaturas académicas y grises, y entrevistas con informantes clave (como usted). Con su consentimiento, se le hará una serie de preguntas a las cuales podrá responder (o no) a su discreción. Si está de acuerdo y con su consentimiento, la entrevista se grabará en un dispositivo de grabación digital y se tomarán notas escritas a mano. Puede elegir ser identificado en la investigación o permanecer completamente en el anonimato; la elección depende totalmente de usted. También tiene derecho a retirarse del estudio, si así lo desea, y puede indicarlo por correo electrónico al investigador en la dirección que se proporciona a continuación.

1. **Para qué se utilizará mi información?**

Tenemos la intención de utilizar nuestros hallazgos para redactar resultados académicos (como artículos de revistas y publicaciones) que ayuden a comprender las politicas gubermentales y la repuesta del brote de COVID-19. Nuestros datos se almacenarán en los servidores XXX protegidos por contraseña durante un período no superior a siete (7) años, después de lo cual se borrarán digitalmente. Si se toman notas escritas a mano, el equipo de XXX las escaneará y cargará en la misma carpeta y las notas originales serán destruidas

1. **Cuánto tiempo tomará la investigación?**

Si acepta participar, esperamos que la entrevista normalmente no dure más de 60 minutos, pero Vd. también puede solicitar una duración más corta.

1. **Puedo retirarme de la investigación?**

Participar en esta investigación es completamente voluntario: no tiene la obligación de dar su consentimiento y, si lo hace, puede retirarse hasta un mes despues de participar sin afectar su relación con XX o con cualquiera de los investigadores involucrados. Puede interrumpir la entrevista en cualquier momento si no desea continuar. Si se retira de la investigación, no retendremos la información que ha proporcionado hasta el momento, a menos que esté feliz de que lo hagamos. Después de la publicación de cualquier hallazgo de la investigación, no será posible retirar su participación.

1. **Alguien más conocerá los resultados?**

Los registros completos de esta investigación se mantendrán de manera confidencial, y sólo el equipo de investigación tendrá acceso a archivos escritos y cintas de audio. Podemos usar un servicio de traducción si es necesario, pero este será un proveedor de XX que puede garantizar la confidencialidad. Todos los archivos y notas digitales recibirán códigos y se almacenarán por separado de cualquier nombre u otra información directamente identificable. El análisis y los resultados de la investigación se harán públicos a través de una variedad de medios (que incluyen, entre otros, presentaciones de conferencias, publicaciones en blogs, revistas revisadas por pares y reuniones informativas sobre políticas). Sin embargo, su nombre puede anonimizarse de cualquier hallazgo directo o cita si lo indica en el formulario de consentimiento.

1. **Tendré algún beneficio de la investigación?**

No podemos y no garantizamos ni prometemos que recibirá beneficios personales de la investigación, pero esperamos que este trabajo contribuya a una comprensión más amplia de las politicas gubermentales de COVID-19 y la interseccion con el tema de genero.

1. **Puedo informar a otras personas acerca de la investigación**

Sí, la decisión depende totalmente de usted.

1. **Qué sucede si necesito más información sobre la investigación o mi participación en ella**

Si desea obtener más información en cualquier momento, no dude en ponerse en contacto con XXXX

Para solicitar una copia de los datos que tenemos sobre usted, contáctese con: [XXXX](mailto:glpd.info.rights@lse.ac.uk)

1. **Qué sucede si tengo una queja o alguna preocupación?**

Si tiene alguna inquietud o queja sobre la realización de esta investigación, comuníquese con el Gerente de Gobernanza de Investigación de XXXX

Esta hoja de información es para Usted. Si le gustaría participar en la investigación, le rogamos firmar o acordar verbalmente el formulario de consentimiento adjunto.

**Gendered Policies to COVID-19**

**PARTICIPANT INFORMATION STATEMENT**

1. **What is the study about?**

You are invited to participate in a study that examines the gender-based social distancing policies implemented by Panama, Peru and latterly Bogota. On Mondays, Wednesdays and Fridays, women are allowed to leave their homes while on Tuesdays, Thursdays and Saturdays only men can. Whilst mortality of COVID-19 is demonstrating more men are dying, we know women disproportionately experience downstream effects of the outbreak; with increased domestic work during self-isolation, increased childcare if schools are shut and are the most at risk of economic insecurity owing to precarious employment contracts. The Panama and Peru policies provide a unique opportunity to examine another potential driver: increased risk of human-to-human transmission due to differences in social distancing practices, and secondary effects of this gendered policy.

1. **Who is carrying out the study?**

The study is being led by researchers at XXX. The research project has undergone ethical review in accordance with the research ethics policy and procedures of the XXX

1. **What does the study involve?**

For our qualitative study we are undertaking a review of academic and grey literatures, and interviews with key informants (such as yourself). With your consent, you will be asked a series of questions that you may choose to answer (or not) at your discretion. If you agree and with your consent, the interview will be recorded on a digital recording device and hand-written notes will be taken. You may choose to be identified in the research, or remain completely anonymous – the choice is entirely up to you. You also retain the right to withdraw from the study, should you wish to do so, and can indicate this via emailing the researcher at the address provided below.

1. **What will my information be used for?**

We intend to use our findings to write academic outputs (such as journal articles and publications) that help to understand -government policies responding to COVID-19 and the impact different policies have on particular communities. Our data will be stored on the password-protected XXX servers for a period not exceeding seven (7) years, after which time they will be digitally erased. If hand-written notes are taken, these will be scanned and uploaded to the same folder by the XXX team and the original notes destroyed.

1. **How much time will the study take?**

If you agree to participate we expect the interview to not normally last more than 60 minutes, but you also can request a shorter duration.

1. **Can I withdraw from the study?**

Participating in this study is completely voluntary - you are not under any obligation to consent and - if you do consent - you can withdraw your participation without affecting your relationship with the XXX or any of the researchers involved up to 1 month after your interview. You may stop the interview at any time if you do not wish to continue. If you withdraw from the study within 1 month, we will not retain the information you have given thus far unless you are happy for us to do so. After publication of any findings from the research, it will not be possible to withdraw your participation.

1. **Will anyone else know the results?**

The full records of this study will be kept confidentially, with only the research team having access to written files and audio tapes. We may use a translation service if necessary, but if so they would be asked to sign a confidentiality agreement. All digital files and notes will be given codes and stored separately from any names or other directly identifiable information. Analysis and results from the research will be made public via a range of mediums (including, but not limited to) conference presentations, blog posts, peer-reviewed journals and policy briefings. However, your name can be anonymised from any direct findings or quotes if you indicate this on the consent form.

1. **Will the study benefit me?**

We cannot and do not guarantee or promise that you will receive any personal benefits from the study, but we hope this work will contribute to a broader understanding of the differential effect of outbreak response to COVID-19

1. **Can I tell other people about the study?**

Yes, the choice is entirely up to you.

1. **What if I require further information about the study or my involvement in it?**

If you would like to know more at any stage, please feel free to contact XXX

To request a copy of the data held about you please contact: [XXX](mailto:glpd.info.rights@lse.ac.uk)

1. **What if I have a complaint or any concerns?**

If you have any concerns or complaints regarding the conduct of this research, please contactXXX

This information sheet is for you to keep. If you are happy to take part in the study, please sign the consent form attached.

**FORMULARIO DE CONSENTIMIENTO DEL PARTICIPANTE**

**Título del proyecto: *Las Politicas de COVID-19 y Genero***

LA PARTICIPACIÓN EN ESTE ESTUDIO DE INVESTIGACIÓN ES VOLUNTARIA

He leído la hoja de información sobre esta investigación y entiendo lo que implica la entrevista propuesta. He podido hacer preguntas sobre el estudio y mis preguntas han sido respondidas satisfactoriamente.

Doy mi consentimiento voluntario para participar en este estudio y entiendo que puedo negarme a responder cualquier pregunta y puedo retirarme del estudio en cualquier momento, sin tener que dar una razón. [S/N]

Estoy de acuerdo con que la entrevista sea grabada en audio [S/N]

Entiendo que la información que proporciono se utilizará para la publicación de la investigación, y la información se anonimizará [S/N]

*por favor marque (sólo) una casilla según corresponda*

Estoy de acuerdo en que mi información pueda citarse anónimamente en los resultados de la investigación [S/N]

Entiendo que cualquier información personal que pueda identificarme, como mi nombre y dirección, se mantendrá confidencial y no se compartirá con nadie más allá del equipo de investigación. [S/N]

Doy permiso para que los investigadores retengan mi información anónima proporcionada en la entrevista para que pueda ser utilizada en futuras investigaciones. [S / N]

Nombre del participante Fecha Firma

Investigador Fecha Firma

**PARTICIPANT CONSENT FORM**

**Title of Project: Gendered Policies to COVID-19**

**Researchers:** XXX

PARTICIPATION IN THIS RESEARCH STUDY IS VOLUNTARY

I have read the information sheet concerning this research and I understand what is involved in the interview proposed. I have been able to ask questions about the study and my questions have been answered to my satisfaction.

I consent voluntarily to be a participant in this study and understand that I can refuse to answer any questions and I can withdraw from the study at any time, without having to give a reason. [Y/N]

I agree to the interview being audio recorded [Y/N]

I understand that the information I provide will be used for research publication, and the information will be anonymised [Y/N]

*please tick (only) one box as appropriate*

I agree that my information can be quoted anonymously in research outputs [Y/N]

I agree that my real name can be used for quotes [Y/N]

I understand that any personal information that can identify me, such as my name and address will be kept confidentially and not shared with anyone beyond the research team. [Y/N]

I give permission for the anonymised data to be deposited in a data archive so that it can be used for future research. [Y/N]

Name of Participant Date Signature

Researcher Date Signature
