# Supplementary material for: Explicitly sexing health security: analysing the downstream effects of Panama’s sex-segregated COVID-19 disease control policy
Source: Health Policy Plan. 2022 Jan 27;37(6):728–36. doi: 10.1093/heapol/czac006 (PMC8807319; doi:10.1093/heapol/czac006)
Supplement: czac006_Supp [file czac006_supp.zip › Annex 2.pdf]

PREGUNTAS / QUESTIONS

OBSERVACIONES GENERALES: General Assumptions

- Considero importante establecer de común acuerdo un objetivo de todas las preguntas para iniciar las entrevistas con dicho objetivo. Por ejemplo: Conocer el impacto de la politica de transito en la socided Panemena. *It is impotant to establish a clear objective with all the questions and to start the interview by stating this onjective. For exmple: to understand the impact of the sex-segregated mobilty policy on Panamanian Society.*

- Hay preguntas que estimo están muy generales y eso puede dificultar la recogida de la información. Sugiero hacerlas un poco más específicas. *There are some questions which are more general, and these can be difficilt to elicit information, if the broad questions do not work, use more specific line of questioning.*

1. Puede contarnos sobre su organización y a quién representa? *Can you tell us about the organisation that you represemt*

Conocimiento de COVID + Vulnerabilidad a la infección *Knoweldge of COVID and Risk of Infection*

2. ¿Piensas que esa respuesta de COVID-19 podría tener efectos diferenciales en diferentes grupos? *Do you think that the resposne to COVID-19 could have differing effects on different groups?*
3. Cómo está afectando este brote a quienes representas? *How is the outbreak affecting those you represent?*

Políticas, Instituciones, Opinión Pública *Policy, Institutions and Public Opinion*

4. Qué piensas de la respuesta del gobierno a la crisis? *What do you think of the government's response to the crisis?*

5. ¿Qué opinas de la política del gobierno que separa los días de tránsito de las personas de acuerdo con el sexo? *What is your opinion of the government's policy to separate people's public mobility by sex?*

6. Como ha afectado al grupo que representas? *How has this affected the group that you represent?*

7. ¿Cuál crees que ha sido el rol de las mujeres durante esta crisis? *What has been the role of women during this crisis?*

8. ¿Cuál crees que ha sido el rol de los hombres durante esta crisis? *What has been the role of men during this crisis?*

9. ¿Crees que las mujeres han sido tomadas en cuenta en el proceso de la creación de políticas públicas durante el covid-19? *Do you think that women have been involved in the development of public policy during COVID-19?*

10. ¿Crees que la respuesta del gobierno considera el impacto de la crisis en las mujeres? *Do you tink that the governmetn response is consdiering the impact of the crisis on women?*

11. Cómo se ha presentado el género o los hombres / mujeres en los medios durante el crisis? *How have the media presented gender/men/women during the crisis?*

12. ¿Piensas que el gobierno ha estado haciendo un buen trabajo en comparación con lo que sabes de los otros países? *Do you think that the government is doing a good job in comparison with that of what you know about the response in other countries?*

13. Crees que se han considerado en las políticas de mitigación de la crisis por el covid- a los siguientes grupos: *Do you think that the following groups have been consdiered in the policies introduced to mititgate the COVID-19 crisis:*
   a) Personas indígenas *Indigenous Groups*
   b) Personas migrantes, desplazadas y refugiadas *Migrants, displaced Populations and refugees*
   c) Personas LGBTI *LGBTI Groups*
   d) Personas afrodescendientes *Black communities*
   e) Personas en extrema pobreza *People in extreme poverty*
   f) Personas con discapacidad *Disabled people*
   g) Personas que viven con VIH *People living with HIV*
   h) Personas adultas mayores *Elderly people*

13. Cómo le gustaría ver cambiar la respuesta del gobierno? *How would you like to see changes to the governmetn resposne?*

Distribución del trabajo *Distribution of work*

14. Qué ha estado haciendo su organización durante la crisis para responder a la política del gobierno? *What is your organisation doing during the crisis to respond to government policy?*

Normas, valores *Norms and values*

15. Cuáles son los principios rectores que guían la respuesta suya a COVID-19 y la repuesta gubermental? *What are the key principles which are guiding your response to COVID-19 and the government response?*

Al final: *Finally*

16. Tiene alguna otra idea sobre los impactos del brote de COVID-19? *Do you have any other thoughts on the impacts of the COVID-19 outbreak?*
17. Hay alguien más con quien creas que necesitamos hablar? *Is there anyone else that you suggest we speak to?*
